# Prediction of High-Grade Clear Cell Renal Cell Carcinoma Based on Plasma mRNA Profiles in Patients with Localized Pathologic T1N0M0 Stage Disease

**DOI:** 10.3390/cancers12051182

**Published:** 2020-05-07

**Authors:** Jee Soo Park, Hyo Jung Lee, Ahmad Almujalhem, Hatem Hamed Althubiany, Alqahatani Ali A, Won Sik Jang, Jongchan Kim, Seung Hwan Lee, Koon Ho Rha, Won Sik Ham

**Affiliations:** 1 Department of Urology and Urological Science Institute, Yonsei University College of Medicine, Seoul 03722, Korea; sampark@yuhs.ac (J.S.P.); q8341@yuhs.ac (H.J.L.); ahmadalmujalhem@gmail.com (A.A.); dr.alialqahtani85@gmail.com (A.A.A.); sindakjang@yuhs.ac (W.S.J.); lumpakcef@yuhs.ac (J.K.); leeseh@yuhs.ac (S.H.L.); khrha@yuhs.ac (K.H.R.); 2 Department of Urology, Imam Abdulrahman Bin Faisal University, Dammam 31441, Saudi Arabia; dr.h.al-thubiany@hotmail.com

**Keywords:** biomarker, clear cell renal cell carcinoma, high-grade, plasma, mRNA profile

## Abstract

A high nuclear grade is crucial to predicting tumor recurrence and metastasis in clear cell renal cell carcinomas (ccRCCs). We aimed to compare the mRNA profiles of tumor tissues and preoperative plasma in patients with localized T1 stage ccRCCs, and to evaluate the potential of the plasma mRNA profile for predicting high-grade ccRCCs. Data from a prospective cohort (*n* = 140) were collected between November 2018 and November 2019. Frozen tumor tissues and plasma were used to measure *PBRM1*, *BAP1*, SET domain-containing 2 (*SETD2*), *KDM5C*, *FOXC2*, *CLIP4*, *AQP1*, *DDX11*, *BAIAP2L1*, and *TMEM38B* mRNA levels, and correlation with the Fuhrman grade was investigated. Multivariate logistic regression analysis revealed significant association between high-grade ccRCC and *SETD2* and *DDX11* mRNA levels in tissues (odds ratio (β) = 0.021, 95% confidence interval (CI): 0.001–0.466, *p* = 0.014; β = 6.116, 95% CI: 1.729–21.631, *p* = 0.005, respectively) and plasma (β = 0.028, 95% CI 0.007–0.119, *p* < 0.001; β = 1.496, 95% CI: 1.187–1.885, *p* = 0.001, respectively). High-grade ccRCC prediction models revealed areas under the curve of 0.997 and 0.971 and diagnostic accuracies of 97.86% and 92.86% for the frozen tissue and plasma, respectively. *SETD2* and *DDX11* mRNA can serve as non-invasive plasma biomarkers for predicting high-grade ccRCCs. Studies with long follow-ups are needed to validate the prognostic value of these biomarkers in ccRCCs.

## 1. Introduction

Renal cell carcinoma (RCC) is the sixth and tenth most common cancer in men and women worldwide, respectively [1]. The rates of incidence of RCC have increased, owing to the increased efficiency of detection using abdominal imaging [2]. Although incidental masses detected in images are small and localized, up to 17% of patients with RCC present with distant metastases at the time of diagnosis, and one-third of patients relapse after surgery [3,4]. Numerous studies have focused on the identification of molecular biomarkers for the prognosis of small RCCs or pT1 RCCs (tumors ≤ 7 cm). Among the various treatment options available to clinicians, ranging from surgical resection to non-surgical approaches, biomarkers are essential for the therapeutic management of RCC; however, an accurate and reliable prognostic indicator remains to be definitively identified [5,6].

Mutations of several tumor suppressor genes (polybromo 1 (*PBRM1*), BRCA1-associated 1 (*BAP1*), and SET domain-containing 2 (*SETD2*)) are associated with clear cell RCC (ccRCC) tumorigenesis [7]. *PBRM1* and *BAP1* mutations are largely mutually exclusive, where mutations of *BAP1* mutations are significantly associated with high-grade, high-stage tumors that result in low survival [8]. Furthermore, mutations of lysine-specific demethylase 5C (*KDM5C*) are associated with poor oncological outcomes [9]. Our previous studies have demonstrated that forkhead box protein C2 (*FOXC2*) and cytoskeleton-associated, protein-glycine (CAP-Gly)-rich, domain-containing linker protein family member 4 (*CLIP4*) mutations were associated with early-stage ccRCC, and a synchronous metastasis [10] and validation study confirmed that *PBRM1*, *BAP1*, and *FOXC2* were shown to be significantly associated with aggressive early-stage ccRCC through target sequencing and immunohistochemistry [5]. In our latest study, we have identified new candidate genes (*AQP1, DDX11, BAIAP2L1*, and *TMEM38B*) by RNA sequencing of formalin-fixed, paraffin-embedded tissues and validating in frozen tissues [11].

Conventional needle biopsies are invasive and subject to procedural complications. Up to one in six biopsies have been reported to be associated with a definite risk [12], and renal mass biopsy (RMB) has shown a relatively high histologic concordance rate, but low nuclear grade concordance rate [13,14,15]. A blood-based test, also known as a liquid biopsy, offers a potential alternative to invasive techniques [16]. In particular, liquid biopsies for circulating tumor cells or circulating tumor DNA (ctDNA) derived from the cell-free DNA (cfDNA) of tumor cells represent a promising method; however, few studies have focused on cfDNA or ctDNA analysis for RCC [17,18]. Considering the lack of satisfactory blood-based markers for ccRCC, there is an urgent need to identify new biomarkers [6,17,18]. Moreover, currently, there exist no panels for cfDNA or ctDNA profiling, and it is comparatively expensive and time-consuming, thus limiting the widespread clinical application of liquid biopsies.

To help resolve these issues, this study aims to evaluate the potential of plasma mRNA profiles in predicting high-grade pT1N0M0 ccRCCs. We compared the mRNA profiles of tumor tissues and plasma samples from patients to determine a correlation between mRNA levels and high nuclear grade (Fuhrman grades 3 and 4), one of the most important factors for predicting tumor recurrence and metastasis after surgery [6]. For the selection of genes, we used the most renown genes that have been identified and validated in our studies and other studies by several research teams. No other studies have been performed on investigating the expression levels of identified genes in plasma. We expect that plasma mRNA profiling could become an inexpensive approach applicable for clinical practice.

## 2. Results

### 2.1. Baseline Characteristics

The clinicopathologic features of patients are listed in Table 1. The mean tumor size was 3.0 ± 1.5 cm. The patient group was primarily comprised of Fuhrman grade 2 (45.0%) and 3 (41.4%) tumors.

### 2.2. Expression of Target Genes in Frozen Tissue and Plasma

**Figure 1:**
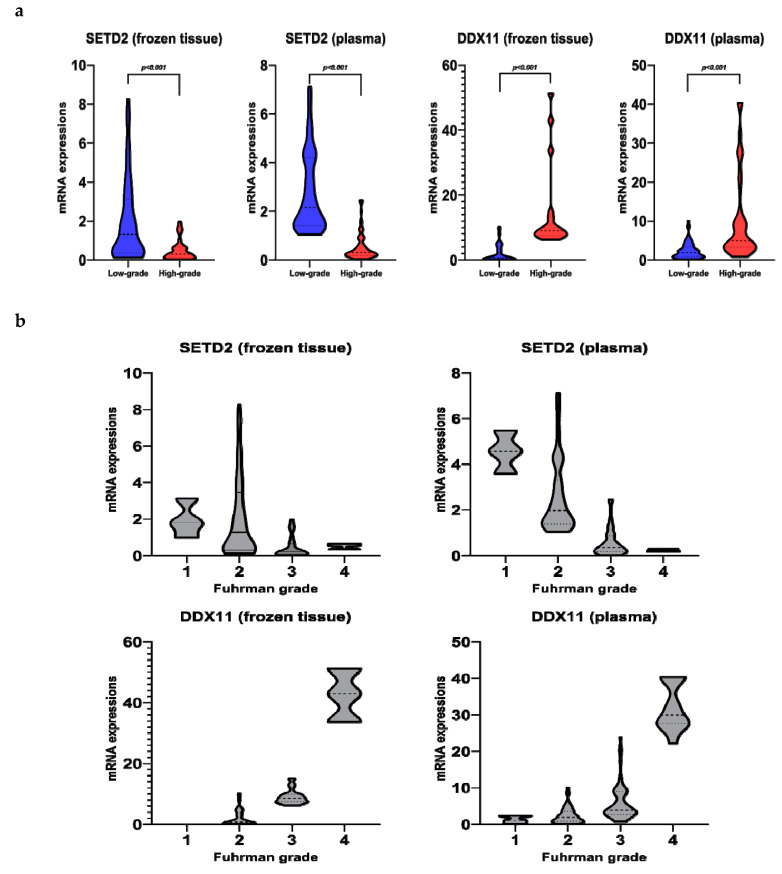
*SETD2* and *DDX11* mRNA levels in frozen tissue and plasma. (**a**) Low-grade vs. high-grade; (**b**) Fuhrman grades.

**Figure 2:**
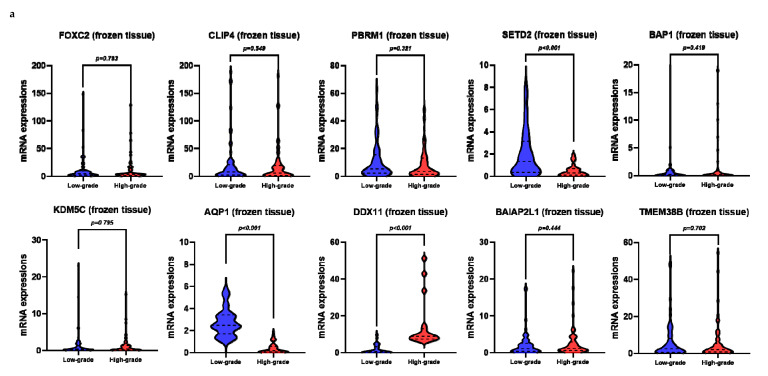

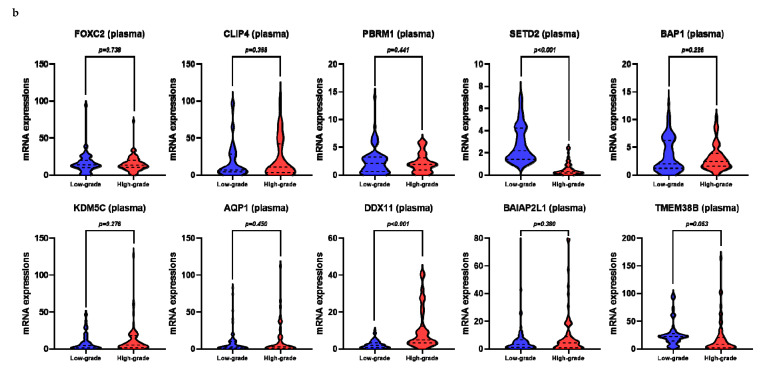
mRNA levels of target genes in low-grade vs. high-grade clear cell renal cell carcinoma (ccRCC): (**a**) frozen tissue; (**b**) plasma.

Using univariate analysis, we observed that the relative mRNA levels of *SETD2*, *AQP1*, and *DDX11* in the frozen tissue significantly correlated with high-grade ccRCC (Figure 1a, Figure 2, and Table S1). Univariate logistic regression analysis revealed that *SETD2*, *AQP1*, and *DDX11* levels were significantly associated with high-grade ccRCC (Table 2). Among these three genes, we only included those that were significantly associated in the plasma samples, in order to effectively compare the performance of prediction models, including the same variables for both frozen tissue and plasma samples. Multivariate logistic regression analyses revealed that *SETD2* and *DDX11* were significantly associated with high-grade ccRCC for the frozen tissues and plasma samples (Table 2).

### 2.3. Correlation between mRNA Levels in the Frozen Tissue and Plasma

*SETD2* mRNA levels in the frozen tissue positively correlated with its plasma levels (Pearson’s correlation coefficient (*r*) = 0.512, *p* < 0.001). Similar results were obtained for *DDX11* (*r* = 0.896, *p* < 0.001).

### 2.4. SETD2 and DDX11 Levels in Frozen Tissue and Plasma, in Accordance with the Fuhrman Grade

The levels of *SETD2* mRNA in the plasma decreased with an increase in the Fuhrman grade, displaying statistically significant differences between grades (Figure 1b and Table S2). However, the levels of *SETD2* mRNA in the frozen tissue did not adhere to overall trends of decrease in *SETD2* mRNA levels as Fuhrman grade increased, only showing statistical differences between grades 2 and 3 and grades 2 and 4. In contrast, *DDX11* levels increased with an increase in the Fuhrman grade of the frozen tissue and plasma samples. Moreover, excluding grades 1 and 2, the difference in the levels was significant among all other grades (Table S2). We further verified the expressions of *SETD2* and *DDX11* from The Cancer Genome Atlas (TCGA) ccRCC database using UALCAN (Figure S1). The TCGA database showed similar expressions trend, according to Fuhrman grades, for both *SETD2* and *DDX11*. *SETD2* expression levels in normal kidney tissue were higher than those in ccRCC; in addition, those in ccRCCs decreased as the Fuhrman grades increased, while *DDX11* levels were the lowest at normal kidney tissue and increased as the Fuhrman grades increased.

### 2.5. Prediction Model Comparison

**Figure 3:**
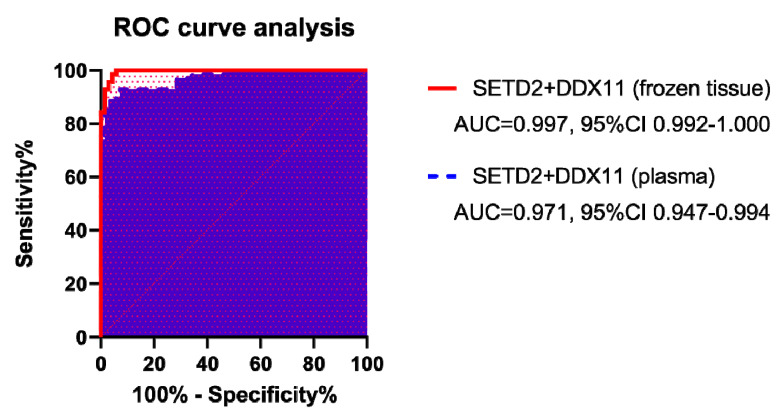
Receiver operating characteristic curves for the prediction models of high-grade clear cell renal cell carcinoma. The area under the curve was 0.997 (95% confidence interval (CI): 0.992–1.000) for *SETD2* and *DDX11* mRNA in frozen tissue, and 0.971 (95% CI: 0.947–0.994) in plasma.

Prediction models based on the mRNA levels of *SETD2* and *DDX11* in frozen tissue presented an area under the curve (AUC) of 0.997 and an accuracy of 97.86%; similarly, those based on plasma mRNA levels of these markers presented an AUC and accuracy of 0.971 and 92.86%, respectively (Table 3 and Figure 3). Although both prediction models performed well, those based on mRNA levels in the frozen tissue were significantly better (*p* = 0.031). As an independent factor, *DDX11* in the frozen tissue gave a better prediction model than *SETD2* in frozen tissue, while *SETD2* in plasma gave a better prediction model than *DDX11* in plasma.

## 3. Discussion

In this study, we identified plasma mRNA levels of *SETD2* and *DDX11* as candidate markers for predicting the prognosis of ccRCC, and developed prediction models for high-grade ccRCC based on the mRNA levels of *SETD2* and *DDX11* in frozen tissues and plasma samples. To the best of our knowledge, this is the first study on the comparison between mRNA profiles of representative genes in plasma and frozen tissues in localized pathological T1N0M0 stage ccRCC. Our findings suggest the use of plasma mRNA markers for predicting high-grade ccRCC, as a means of supplementing the low-nuclear-grade concordance rate of conventional RMB.

*SETD2* and *DDX11* mRNA levels in the plasma positively correlated with those in the frozen tissue. The advantages of using plasma over conventional invasive biopsies include decreased risk due to testing and ease of repetitive testing. Moreover, the concordance between the expression in the tissue and plasma indicates that the plasma mRNA profiles of *SETD2* and *DDX11* can be used as reliable genetic markers for tumors.

The models based on tissue and plasma expression performed well in predicting high-grade ccRCCs, the former being significantly better. Notably, our frozen tissue expression model was based on surgical specimens. To best compare the performance of the two models, the plasma expression should be compared with that in percutaneous RMB. RMB has been historically limited, owing to concerns associated with its high non-diagnostic rate and complications, including biopsy tract seeding; however, recent studies have reported improved safety and diagnostic accuracy of RMB in detecting malignant tumors and histological subtyping [14,15,19]. Nevertheless, RMB continues to have poor accuracy for assessing the Fuhrman grade, a key determinant of the biological potential of ccRCC, as well as the disease stage that is used in multiple prognostic models; moreover, nuclear grade heterogeneity is a substantial issue in RMB [19]. We believe that the use of RMB in conjunction with plasma mRNA levels of *SETD2* and *DDX11* can improve the accuracy of assessing the Fuhrman grade. Further studies are needed to confirm the predictive ability of the combination of mRNA levels in plasma and tissues in RMB samples.

*DDX11* is an important gene for predicting tumor aggressiveness based on the Fuhrman grade. Bhattacharya et al. [20] had reported that *DDX11* inhibition decreased the rate of proliferation and induced apoptosis in melanoma cells. Moreover, *DDX11* was significantly upregulated and associated with a poor prognosis in patients with lung adenocarcinoma [21]. Consistent with these findings, we observed that *DDX11* expression increased with the Fuhrman grade in frozen tissue and plasma samples.

Furthermore, *SETD2* was significantly associated with high-grade ccRCC in frozen tissues and plasma, and *SETD2* levels decreased with an increase in the Fuhrman grade. *SETD2*, which encodes a histone methyltransferase, is a novel tumor suppressor gene in ccRCC. The Cancer Genome Atlas (TCGA) revealed that alterations in DNA methylation correlate with *SETD2* mutations [22]. *SETD2* mutations have been reported at rates of 11.6% and 7.4% in TCGA and Memorial Sloan-Kettering Cancer Center cohorts, respectively [23], and Liu et al. [24] reported a rate of deficiency of *SETD2* of 34.1% in a multicenter study. *SETD2* mutation has been found to be associated with adverse oncological outcomes in our previous study and other multicenter studies, thereby suggesting a general role of *SETD2* in disease progression [5,24]. Consistent with our findings, lower *SETD2* mRNA levels have been reported in breast cancer tissues, thereby linking decreased *SETD2* mRNA levels to tumorigenesis [25].

*AQP1* was reported to be an important gene associated with high-grade ccRCC in frozen tissues, although no significance has been reported for its plasma levels. Huang et al. [26] demonstrated that *AQP1* levels were significantly higher in patients whose tumors were smaller, of lower grade, or either a lower stage or lacking microvascular invasion. Furthermore, higher *AQP1* levels were associated with a better prognosis of cancer-specific and cancer-free survival. In accordance with these findings, we found similar trends for *AQP1* expression, wherein *AQP1* expression levels were higher in patients with low-grade ccRCC.

However, this study has a few limitations. First, the study period was only one year. Therefore, the follow-up period was too short to evaluate the significance of biomarkers with respect to ccRCC prognostic factors, such as recurrence, cancer-specific death, or survival. Furthermore, none of the 140 patients included in this study developed recurrence, metastasis, or cancer-specific death. As a high nuclear grade is currently the most important prognostic factor for predicting tumor recurrence and metastasis after surgery [6], we evaluated the correlation between the expression of biomarkers and a high nuclear grade. Second, due to the uneven number of patients in each Fuhrman grade, statistical significances were not noted between some grade groups, and expression levels between some grade groups did not adhere to overall mRNA expression trends of low- and high-grade ccRCC. Recently, the World Health Organization/International Society of Urological Pathology (WHO/ISUP) grading has been replacing Fuhrman grading, which previously had been used widely, since the Fuhrman grading requires simultaneous assessment of three different parameters, resulting in poor interobserver reproducibility [27]. In our institution, the nuclear grade was classified according to both the Fuhrman and the ISUP grading systems, and our analysis on ISUP grading results were similar (Tables S3 and S4). Moreover, since our study referred to the previous studies that analyzed the genetic profiles according to Fuhrman grading, we tried to adhere to Fuhrman grading in this study. In a future study, we are planning to match the number of patients in each grade group by using ISUP grading, which is more widely used. Third, the intratumoral heterogeneity of primary tumors is a substantial problem, even in small renal masses [28]. Intratumoral heterogeneity is known to cause sampling bias in conventional sampling methods, such as needle biopsies, leading to false-negative results or suboptimal therapy selection, due to low grading accuracy [13,14,15]. Liquid biopsies can supplement this limitation, and our findings highlight plasma *SETD2* and *DDX11* mRNA levels as promising candidates for predicting high-grade ccRCC with this approach. Although the relative quantification approach was used in this study, the absolute quantification approach would provide more exact values, which is helpful in real clinical practices. Therefore, the use of absolute quantification of plasma *SETD2* and *DDX11* levels should be investigated in a future study. Moreover, by including the analysis of the normal kidney tissue and healthy donor plasma, a future study could suggest the use of the expressions of *SETD2* and *DDX11* as the tool for diagnosis of early ccRCCs. Furthermore, the discrepancy between the different validity of *SETD2* and *DDX11*—which is that *DDX11* in frozen tissue gives a better prediction model than *SETD2*, while the opposite results occur for *SETD2* in plasma—was reported in our study. Although we do not clearly understand the underlying mechanisms, the distortion of biomarkers by different release processes in tissue and plasma could be explained by the inclusion of the normal kidney tissue and healthy donor plasma. Therefore, in our coming projects, we have included the analysis of normal kidney tissue and healthy donor plasma, which will be reported in a future study.

We are currently conducting clinical trials for investigating the use of ctDNA as a marker in urological malignancies (ClinicalTrials.gov identifier: NCT04197414). We believe that this study will serve as the cornerstone for the initiation of the liquid biopsy era in the context of ccRCC diagnosis and management.

## 4. Materials and Methods 

### 4.1. Patients and Tissues

A prospective cohort (ClinicalTrials.gov identifier: NCT03694912) was selected between November 2018 and November 2019; it included patients with pT1N0M0 ccRCC (≤7 cm). All subjects gave their informed consent for inclusion before they participated in the study. The study was approved by the Institutional Review Board of the Yonsei University Health System (approval no: 4-2018-0753).

In total, 140 patients with ccRCC (≤7 cm), who were treated via nephrectomy alone and for whom frozen tumor tissue and matched preoperative plasma were available, were included in the study. Patients not presenting typical characteristics of ccRCC, those receiving neoadjuvant or adjuvant systemic therapy, those with a history of inherited von Hippel–Lindau disease, or those with synchronous/metachronous bilateral RCC were excluded. Patients with no or very little tumor tissue (less than 5% of the area occupied by invasive cancer cells), insufficient RNA, or inadequate RNA quality were also excluded.

Clinicopathologic data, including age, sex, body mass index, and tumor size were recorded for each patient. The histological subtype was assessed in accordance with the 2016 World Health Organization Renal Neoplasms guidelines [29], and invasion (perinephric/sinus fat or microscopic vascular invasion), and lymph node involvement were evaluated in accordance with the guidelines of the 2010 American Joint Committee on Cancer [30]. Grading was based on Fuhrman grading [31] and the WHO/ISUP grading system [32]. Low-grade ccRCC consists of Fuhrman or WHO/ISUP grades I and II, whereas grades III and IV constitute high-grade ccRCC. Diameters of the primary tumors were obtained via imaging.

We analyzed the expression of six genes (*PBRM1*, *BAP1*, *SETD2*, *KDM5C*, *FOXC2*, and *CLIP4*) reportedly associated with ccRCC, and four genes (*AQP1*, *DDX11*, *BAIAP2L1*, and *TMEM38B*) from our previous RNA-seq analysis of aggressive ccRCC at the clinical T1 stage [11].

### 4.2. Processing of Blood Samples

Peripheral blood was collected from each participant, aliquoted into ethylenediaminetetraacetic acid-containing tubes, and centrifuged at 1600× *g* for 10 min at 4 °C. Plasma was carefully transferred to fresh tubes and further centrifuged at 4000× *g* for 10 min at 4 °C. Plasma samples were then stored at −80 °C until further analyses.

### 4.3. RNA Extraction and Reverse Transcription-Quantitative Polymerase Chain Reaction (RT-qPCR)

Total RNA was extracted from frozen tissue and plasma samples using TRIzol (Ambion, Life Technologies, Carlsbad, CA, USA). In general, RNA isolated from 1 mL of plasma was dissolved in 20 μL of diethyl-pyrocarbonate (DEPC)-treated water. The quantity and quality of RNA was assessed using a Nanodrop spectrophotometer (NanoDrop ND-1000, Thermo Scientific, Wilmington, DE, USA). One microgram of each sample was reverse-transcribed into first-strand cDNA using the iNtRon Maxime RT PreMix (iNtRON catalog no. 25082), in accordance with the manufacturer’s protocol. Then qPCR was performed using the Power SYBR Green Master Mix (Thermo Fisher, catalog no. A25742, Waltham, MA, USA) in a 10 μL reaction volume comprised of 5 μL of SYBR Green master PCR mix, 1 μL of forward and reverse primer (10 pmol), 1 μL of diluted cDNA template, and sterile distilled water. Conditions for amplification were as follows: initial denaturation at 95 °C for 10 min, 40 cycles of denaturation at 95 °C for 15 s, annealing at 58 °C for 60 s, elongation at 72 °C for 60 s, and final elongation at 72 °C for 5 min. qPCR was performed on the ABI StepOnePlus Real-Time PCR System (Applied Biosystems, Foster City, CA, USA). All quantifications were carried out using *GAPDH* as a reference gene to normalize relative target gene expression levels. *GAPDH* levels were constant across samples, with no significant variations in either the frozen tissues or the plasma, respectively, although *GAPDH* levels were different between them. The PCR primer sequences are presented in Table S5. Relative gene expression was analyzed using the 2^−ΔΔCT^ method. The transcript copy number of each genes was normalized to the *GAPDH* transcript copy number for each sample. RT-qPCR was performed in triplicates or more, and the results were analyzed in an investigator-blinded manner.

### 4.4. UALCAN Analysis

UALCAN [33], an online tool used to analyze gene expression data from The Cancer Genome Atlas (TCGA) database (including 72 normal kidney tissues and 533 primary tumors), was used to validate the mRNA expressions of SETD2 and DDX11 according to Fuhrman grades. A *p*-value less than 0.05 was considered to indicate a statistically significant difference [34].

### 4.5. Statistical Analyses

Data have been presented as the mean ± standard deviation or median (interquartile range) for continuous variables, and as a percentage for categorical variables. For univariate analysis, *t*-test and univariate logistic regression analysis were used to compare continuous variables. Multivariate logistic regression analyses were performed for factors that were significantly associated with high-grade ccRCCs in both frozen tissue and plasma in univariate logistic regression analyses. Correlation between the mRNA levels of *SETD2* and *DDX11* in frozen tissue and plasma were determined using Pearson’s correlation analysis. Levels of *SETD2* and *DDX11* mRNA, in accordance with the Fuhrman or WHO/ISUP grade, were analyzed using one-way analysis of variance, followed by the Bonferroni post-hoc test. SPSS software version 23.0 (IBM Corp., Armonk, NY, USA) was used for all statistical analyses. All statistical tests were two-tailed, and a *p-*value less than 0.05 was considered significant. GraphPad Prism software (version 8.0, GraphPad Software, Inc., La Jolla, CA, USA) was used for generating the graphs.

### 4.6. Prediction Model Comparison

The accuracy, sensitivity, specificity, positive predictive value (PPV), and negative predictive value (NPV) of the prediction models were calculated. The receiver operating characteristic curves and area under the curve (AUC) were used to examine and compare the performance of the prediction models for high-grade ccRCC using the R package (R Development Core Team, 2010). 

## 5. Conclusions

In summary, we evaluated the mRNA profiles of candidate genes in the frozen tissues and plasma of patients with localized T1N0M0 ccRCC. *SETD2* and *DDX11* mRNA levels in frozen tissues and plasma were significantly associated with high-grade ccRCC. The determination of plasma *SETD2* and *DDX11* mRNA levels in the plasma can be used as a means of supplementing the low-grade accuracy of conventional RMB, by including ease, safety, and the ability to provide more information on the tumor. Moreover, non-invasive plasma biomarkers can complement RMB in the clinical characterization of localized T1 renal masses.

## Figures and Tables

**Table 1:** Clinical and histopathological characteristics.

Characteristic	Clinicopathological Data (*n* = 140)
Sex male/female	97/43 (69.3/30.7%)
Age (years)	56.0 ± 12.3
Mean tumor diameter (cm)	3.0 ± 1.5
Median tumor diameter (cm)	2.8 (1.7–4.0)
Fuhrman grade	
1	7 (5.0%)
2	63 (45.0%)
3	58 (41.4%)
4	12 (8.6%)

Data are presented as mean ± standard deviation or median (interquartile range) for continuous variables, and as a percentage for categorical variables.

**Table 2:** Univariate and multivariate logistic regression analyses of the mRNA levels of target genes associated with high-grade clear cell renal cell carcinoma.

High-Grade ccRCC	Univariate β (95% CI)	*p* ^a^	Multivariate β (95% CI)	*p* ^b^
*FOXC2*	0.998 (0.982–1.014)	0.792		
*CLIP4*	0.997 (0.988–1.007)	0.548		
*PBRM1*	0.987 (0.961–1.013)	0.321		
*SETD2*	0.303 (0.177–0.520)	<0.001	0.021 (0.001–0.466)	0.014
*BAP1*	1.620 (0.488–5.383)	0.431		
*KDM5C*	0.984 (0.876–1.107)	0.794		
*AQP1*	0.012 (0.002–0.077)	<0.001		
*DDX11*	2.625 (1.811–3.805)	<0.001	6.116 (1.729–21.631)	0.005
*BAIAP2L1*	1.039 (0.942–1.146)	0.446		
*TMEM38B*	0.993 (0.959–1.029)	0.700		
*FOXC2*	0.995 (0.967–1.024)	0.736		
*CLIP4*	1.006 (0.993–1.019)	0.367		
*PBRM1*	0.939 (0.800–1.102)	0.440		
*SETD2*	0.045 (0.015–0.132)	<0.001	0.028 (0.007–0.119)	<0.001
*BAP1*	0.926 (0.818–1.048)	0.225		
*KDM5C*	1.013 (0.989–1.037)	0.289		
*AQP1*	1.008 (0.988–1.028)	0.451		
*DDX11*	1.504 (1.255–1.803)	<0.001	1.496 (1.187–1.885)	0.001
*BAIAP2L1*	1.010 (0.988–1.033)	0.384		
*TMEM38B*	0.986 (0.972–1.001)	0.062		

^a^*p*-value calculated using logistic regression for univariate analysis; ^b^*p*-value calculated using logistic regression for multivariate analysis.

**Table 3:** Performance comparison of logistic regression models for prediction of high-grade clear cell renal cell carcinoma.

Included Variables in Models	Sensitivity	Specificity	PPV	NPV	Accuracy (%)	AUC (95% CI)
*SETD2* (frozen tissue)	0.84	0.59	0.84	0.59	71.43%	0.779 (0.704–0.853)
*DDX11* (frozen tissue)	1.00	0.93	1.00	0.93	96.43%	0.964 (0.931–0.997)
*SETD2* + *DDX11* (frozen tissue)	0.97	0.99	0.97	0.99	97.86%	0.997 (0.992–1.000)
*SETD2* (plasma)	0.86	1.00	1.00	0.86	92.86%	0.952 (0.918–0.987)
*DDX11* (plasma)	0.84	0.70	0.84	0.70	77.14%	0.836 (0.771–0.900)
*SETD2* + *DDX11* (plasma)	0.93	0.93	0.93	0.93	92.86%	0.971 (0.947–0.994)

PPV: positive predictive value; NPV: negative predictive value; AUC: area under the curve; CI: confidence interval.

## Supplementary Materials

The following are available online at https://www.mdpi.com/2072-6694/12/5/1182/s1, Table S1: mRNA levels of target genes in low-grade vs. high-grade clear cell renal cell carcinoma. Table S2: *SETD2* and *DDX11* levels in clear cell renal cell carcinomas of different Fuhrman grades. Table S3: Univariate and multivariate logistic regression analyses of the mRNA levels of target genes associated with high-grade clear cell renal cell carcinoma (by International Society of Urological Pathology (ISUP) grading system). Table S4: *SETD2* and *DDX11* levels in clear cell renal cell carcinomas of different ISUP grades. Table S5: PCR primer sequences. Figure S1: Validation of the expression of *SETD2* and *DDX11* in the TCGA database, according to Fuhrman grades.

## References

[1] Siegel R.L., Miller K.D., Jemal A. (2018). Cancer statistics, 2018. CA Cancer J. Clin..

[2] Capitanio U., Bensalah K., Bex A., Boorjian S.A., Bray F., Coleman J., Gore J.L., Sun M., Wood C., Russo P. (2019). Epidemiology of renal cell carcinoma. Eur. Urol..

[3] Capitanio U., Montorsi F. (2016). Renal cancer. Lancet.

[4] Rini B.I., Campbell S.C., Escudier B. (2009). Renal cell carcinoma. Lancet.

[5] Park J.S., Lee H.J., Cho N.H., Kim J., Jang W.S., Heo J.E., Ham W.S. (2019). Risk prediction tool for aggressive tumors in clinical T1 stage clear cell renal cell carcinoma using molecular biomarkers. Comput. Struct. Biotechnol. J..

[6] Suzuki K., Mizuno R., Mikami S., Tanaka N., Kanao K., Kikuchi E., Miyajima A., Nakagawa K., Oya M. (2012). Prognostic significance of high nuclear grade in patients with pathologic T1a renal cell carcinoma. Jpn. J. Clin. Oncol..

[7] Mitchell T.J., Turajilic S., Rowan A., Nicol D., Farmery J.H., O’Brien T., Martincorena I., Tarpey P., Angelopoulos N., Yates L.R. (2018). Timing the landmark events in the evolution of clear cell renal cell cancer: TRACERx Renal. Cell.

[8] Peña-Llopis S., Vega-Rubin-de-Celis S., Liao A., Leng N., Pavía-Jiménez A., Wang S., Yamasaki T., Zhrebker L., Sivanand S., Spence P. (2012). BAP1 loss defines a new class of renal cell carcinoma. Nat. Genet..

[9] Dalgliesh G.L., Furge K., Greenman C., Chen L., Bignell G., Butler A., Davies H., Edkins S., Hardy C., Latimer C. (2010). Systematic sequencing of renal carcinoma reveals inactivation of histone modifying genes. Nature.

[10] Ahn J., Han K.S., Heo J.H., Bang D., Kang Y.H., Jin H.A., Hong S.J., Lee J.H., Ham W.S. (2016). FOXC2 and CLIP4: A potential biomarker for synchronous metastasis of ≤7-cm clear cell renal cell carcinomas. Oncotarget.

[11] Park J.S., Pierorazio P.M., Lee J.H., Lee H.J., Lim Y.S., Jang W.S., Kim J., Lee S.H., Rha K.H., Cho N.H. (2020). Gene expression analysis of aggressive clinical T1 stage clear cell renal cell carcinoma for identifying potential diagnostic and prognostic biomarkers. Cancers.

[12] Overman M.J., Modak J., Kopetz S., Murthy R., Yao J.C., Hicks M.E., Abbruzzese J.L., Tam A.L. (2013). Use of research biopsies in clinical trials: Are risks and benefits adequately discussed?. J. Clin. Oncol..

[13] Caoili E.M., Davenport M.S. (2014). Role of percutaneous needle biopsy for renal masses. Semin Interv. Radiol..

[14] Leveridge M.J., Finelli A., Kachura J.R., Evans A., Chung H., Shiff D.A., Fernandes K., Jewett M.A. (2011). Outcomes of small renal mass needle core biopsy, nondiagnostic percutaneous biopsy, and the role of repeat biopsy. Eur. Urol..

[15] Neuzillet Y., Lechevallier E., Andre M., Daniel L., Coulange C. (2004). Accuracy and clinical role of fine needle percutaneous biopsy with computerized tomography guidance of small (less than 4.0 cm) renal masses. J. Urol..

[16] Wan J.C., Massie C., Garcia-Corbacho J., Mouliere F., Brenton J.D., Caldas C., Pacey S., Baird R., Rosenfeld N. (2017). Liquid biopsies come of age: Towards implementation of circulating tumour DNA. Nat. Rev. Cancer.

[17] Yamamoto Y., Uemura M., Fujita M., Maejima K., Koh Y., Matsushita M., Nakano K., Hayashi Y., Wang C., Ishizuya Y. (2019). Clinical significance of the mutational landscape and fragmentation of circulating tumor DNA in renal cell carcinoma. Cancer Sci..

[18] Pal S.K., Sonpavde G., Agarwal N., Vogelzang N.J., Srinivas S., Haas N.B., Signoretti S., McGregor B.A., Jones J., Lanman R.B. (2017). Evolution of circulating tumor DNA profile from first-line to subsequent therapy in metastatic renal cell carcinoma. Eur. Urol..

[19] Ball M.W., Bezerra S.M., Gorin M.A., Cowan M., Pavlovich C.P., Pierorazio P.M., Netto G.J., Allaf M.E. (2015). Grade heterogeneity in small renal masses: Potential implications for renal mass biopsy. J. Urol..

[20] Bhattacharya C., Wang X., Becker D. (2012). The DEAD/DEAH box helicase; DDX11; is essential for the survival of advanced melanomas. Mol. Cancer.

[21] Li J., Liu L., Liu X., Xu P., Hu Q., Yu Y. (2019). The role of upregulated *DDX11* as a potential prognostic and diagnostic biomarker in lung adenocarcinoma. J. Cancer.

[22] Cancer Genome Atlas Research Network (2013). Comprehensive molecular characterization of clear cell renal cell carcinoma. Nature.

[23] Hakimi A.A., Ostrovnaya I., Reva B., Schultz N., Chen Y.B., Gonen M., Liu H., Takeda S., Voss M.H., Tickoo S.K. (2013). Adverse outcomes in clear cell renal cell carcinoma with mutations of 3p21 epigenetic regulators BAP1 and SETD2: A report by MSKCC and the KIRC TCGA research network. Clin. Cancer Res..

[24] Liu L., Guo R., Zhang X., Liang Y., Kong F., Wang J., Xu Z. (2017). Loss of SETD2; but not H3K36me3; correlates with aggressive clinicopathological features of clear cell renal cell carcinoma patients. Biosci. Trends.

[25] Al Sarakbi W., Sasi W., Jiang W.G., Roberts T., Newbold R.F., Mokbel K. (2009). The mRNA expression of SETD2 in human breast cancer: Correlation with clinico-pathological parameters. BMC Cancer.

[26] Huang Y., Murakami T., Sano F., Kondo K., Nakaigawa N., Kishida T., Kubota Y., Nagashima Y., Yao M. (2009). Expression of aquaporin 1 in primary renal tumors: A prognostic indicator for clear-cell renal cell carcinoma. Eur. Urol..

[27] Dagher J., Delahunt B., Rioux-Leclercq N., Egevad L., Srigley J.R., Coughlin G., Dunglinson N., Gianduzzo T., Kua B., Malone G. (2017). Clear cell renal cell carcinoma: Validation of World Health Organization/International Society of Urological Pathology grading. Histopathology.

[28] Manley B.J., Reznik E., Ghanaat M., Kashan M., Becerra M.F., Casuscelli J., Tennenbaum D., Redzematovic A., Carlo M.I., Sato Y. (2017). Characterizing recurrent and lethal small renal masses in clear cell renal cell carcinoma using recurrent somatic mutations. Urol. Oncol..

[29] Moch H., Humphrey P.A., Ulbright T.M., Reuter V. (2016). WHO Classification of Tumours of the Urinary System and Male Genital Organs.

[30] Edge S.B., Compton C.C. (2010). The American Joint Committee on Cancer: The 7th edition of the AJCC cancer staging manual and the future of TNM. Ann. Surg. Oncol..

[31] Fuhrman S.A., Lasky L.C., Limas C. (1982). Prognostic significance of morphologic parameters in renal cell carcinoma. Am. J. Surg. Pathol..

[32] Delahunt B., Cheville J.C., Martignoni G., Humphrey P.A., Magi-Galluzzi C., McKenney J., Egevad L., Algaba F., Moch H., Grignon D.J. (2013). The International Society of Urological Pathology (ISUP) grading system for renal cell carcinoma and other prognostic parameters. Am. J. Surg. Pathol..

[33] UALCAN. http://ualcan.path.uab.edu.

[34] Lanczky A., Nagy A., Bottai G., Munkácsy G., Szabó A., Santarpia L., Győrffy B. (2016). miRpower: A web-tool to validate survival-associated miRNAs utilizing expression data from 2178 breast cancer patients. Breast Cancer Res Treat..

