# Supplementary material for: Prediction of High-Grade Clear Cell Renal Cell Carcinoma Based on Plasma mRNA Profiles in Patients with Localized Pathologic T1N0M0 Stage Disease
Source: Cancers (Basel). 2020 May 7;12(5):1182. doi: 10.3390/cancers12051182 (PMC7281002; doi:10.3390/cancers12051182)
Supplement: Supplementary file 1 [file cancers-12-01182-s001.pdf]

Supplementary Materials Prediction of High-Grade Clear Cell Renal Cell Carcinoma Based on Plasma mRNA Profiles in Patients with Localized Pathologic T1N0M0 Stage Disease

Jee Soo Park, Hyo Jung Lee, Ahmad Almujalhem, Hatem Hamed Althubiany, Alqahatani Ali A, Won Sik Jang, Jongchan Kim, Seung Hwan Lee, Koon Ho Rha and Won Sik Ham

Table S1. mRNA levels of target genes in low-grade vs. high-grade clear cell renal cell carcinoma

**mRNA level (×100)**	**Low-grade RCC (*N* = 70)**	**High-grade RCC (*N* = 70)**	***p***
FOXC2	10.854 ± 21.300	9.939 ± 19.911	0.793
CLIP4	21.725 ± 39.657	18.091 ± 31.509	0.549
PBRM1	11.071 ± 13.581	8.927 ± 11.808	0.321
SETD2	2.013 ± 2.108	0.477 ± 0.514	<0.001
BAP1	6.473 ± 17.572	10.678 ± 39.708	0.419
KDM5C	1.335 ± 3.328	1.209 ± 2.319	0.795
AQP1	2.733 ± 1.226	0.404 ± 0.476	<0.001
DDX11	1.824 ± 2.624	14.712 ± 13.281	<0.001
BAIAP2L1	2.139 ± 3.139	2.594 ± 3.843	0.444
TMEM38B	6.042 ± 9.580	5.424 ± 9.480	0.702
FOXC2	15.700 ± 12.882	15.039 ± 10.260	0.738
CLIP4	18.697 ± 24.309	22.623 ± 27.090	0.368
PBRM1	2.496 ± 2.470	2.218 ± 1.699	0.441
SETD2	2.772 ± 1.636	0.528 ± 0.578	<0.001
BAP1	3.598 ± 3.099	3.036 ± 2.299	0.226
KDM5C	8.692 ± 11.177	11.552 ± 18.831	0.276
AQP1	8.418 ± 15.349	10.631 ± 18.999	0.450
DDX11	2.284 ± 2.111	10.471 ± 11.220	<0.001
BAIAP2L1	7.349 ± 15.281	9.690 ± 16.146	0.380
TMEM38B	27.100 ± 22.861	18.602 ± 28.460	0.053

Data are shown as mean ± SD values. *p*-values were determined using Student's *t*-test.

Table S2. *SETD2* and *DDX11* levels in clear cell renal cell carcinomas of different Fuhrman grades.

**Fuhrman Grade**	***N***	***SETD2* Expression (*Frozen Tissue*) (mean ± standard deviation)**	***p*-Value between the Two Categories**			
			**G1**	**G2**	**G3**	**G4**
G1	7	1.935 ± 0.883	—	1.000	0.115	0.333
G2	63	2.021 ± 2.207	—	—	<0.001	0.014
G3	58	0.469 ± 0.562	—	—	—	1.000
G4	12	0.515 ± 0.130	—	—	—	—
Fuhrman Grade		*SETD2* expression (*plasma*) (mean ± standard deviation)	*P* value between the two categories			
			G1	G2	G3	G4
G1	7	4.542 ± 0.773	—	<0.001	<0.001	<0.001
G2	63	2.575 ± 1.590	—	—	<0.001	<0.001
G3	58	0.588 ± 0.618	—	—	—	<0.001
G4	12	0.236 ± 0.053	—	—	—	—
Fuhrman Grade		*DDX11* expression (*frozen tissue*) (mean ± standard deviation)	*P* value between the two categories			
			G1	G2	G3	G4
G1	7	0.006 ± 0.002	—	0.671	<0.001	<0.001
G2	63	2.026 ± 2.693	—	—	<0.001	<0.001
G3	58	8.944 ± 2.281	—	—	—	<0.001
G4	12	42.590 ± 7.500	—	—	—	—
Fuhrman Grade		*DDX11* expression (*plasma*) (mean ± standard deviation)	*P* value between the two categories			
			G1	G2	G3	G4
G1	7	1.292 ± 0.999	—	1.000	0.024	<0.001
G2	63	2.394 ± 2.177	—	—	<0.001	<0.001
G3	58	5.987 ± 4.976	—	—	—	<0.001
G4	12	32.141 ± 6.563	—	—	–	–

p-value determined using one-way analysis of variance followed by Bonferroni post-hoc test.

Table S3. Univariate and multivariate logistic regression analyses of the mRNA levels of target genes associated with high-grade clear cell renal cell carcinoma (by ISUP grading system).

High-grade ccRCC	Univariate ß (95% CI)	*p* [a]	Multivariate ß (95% CI)	*p* [b]
FOXC2	0.999 (0.983–1.016)	0.918		
CLIP4	0.997 (0.987–1.007)	0.550		
PBRM1	0.979 (0.951–1.007)	0.143		
SETD2	0.351 (0.206–0.598)	<0.001	0.290 (0.128–0.655)	0.003
BAP1	0.999 (0.988–1.010)	0.841		
KDM5C	0.996 (0.885–1.120)	0.942		
AQP1	0.095 (0.044–0.203)	<0.001	—	—
DDX11	2.049 (1.564–2.685)	<0.001	2.173 (1.545–3.056)	<0.001
BAIAP2L1	1.026 (0.933–1.129)	0.594		
TMEM38B	0.997 (0.962–1.033)	0.852		
FOXC2	0.994 (0.965–1.024)	0.678		
CLIP4	1.005 (0.992–1.018)	0.497		
PBRM1	0.988 (0.843–1.159)	0.884		
SETD2	0.121 (0.058–0.253)	<0.001	0.097 (0.036–0.258)	<0.001
BAP1	0.932 (0.821–1.058)	0.275		
KDM5C	1.023 (0.996–1.049)	0.093		
AQP1	1.013 (0.993–1.034)	0.206		
DDX11	1.777 (1.418–2.227)	<0.001	1.781 (1.326–2.392)	<0.001
BAIAP2L1	1.015 (0.992–1.037)	0.205		
TMEM38B	0.983 (0.968–0.999)	0.040	—	—

[a] *p*-value calculated using logistic regression for univariate analysis; [b] *p*-value calculated using logistic regression for multivariate analysis.

Table S4. *SETD2* and *DDX11* levels in clear cell renal cell carcinomas of different ISUP grades.

**ISUP Grade**	***N***	***SETD2* Expression (*Frozen Tissue*) (mean ± standard deviation)**	***p*-Value between the Two Categories**			
			**G1**	**G2**	**G3**	**G4**
G1	23	1.448 ± 1.036	—	1.000	0.105	0.498
G2	57	1.967 ± 2.323	—	—	<0.001	0.017
G3	47	0.478 ± 0.584	—	—	—	1.000
G4	13	0.488 ± 0.160	—	—	—	—
ISUP Grade		*SETD2* expression (*plasma*) (mean ± standard deviation)	*p* value between the two categories			
			G1	G2	G3	G4
G1	23	3.060 ± 1.482	—	0.098	<0.001	<0.001
G2	57	2.258 ± 1.760	—	—	<0.001	<0.001
G3	47	0.576 ± 0.611	—	—	—	1.000
G4	13	0.371 ± 0.490	—	—	—	—
ISUP Grade		*DDX11* expression (*frozen tissue*) (mean ± standard deviation)	*p* value between the two categories			
			G1	G2	G3	G4
G1	23	1.334 ± 2.353	—	0.691	<0.001	<0.001
G2	57	3.081 ± 3.479	—	—	<0.001	<0.001
G3	47	9.180 ± 2.221	—	—	—	<0.001
G4	13	39.982 ± 11.829	—	—	—	—
ISUP Grade		*DDX11* expression (*plasma*) (mean ± standard deviation)	*p* value between the two categories			
			G1	G2	G3	G4
G1	23	0.679 ± 0.691	—	0.087	<0.001	<0.001
G2	57	2.818 ± 2.020	—	—	<0.001	<0.001
G3	47	6.532 ± 4.389	—	—	—	<0.001
G4	13	31.504 ± 6.691	—	—	–	–

p-value determined using one-way analysis of variance followed by Bonferroni post-hoc test.

Table S5. PCR primer sequences.

**Target Gene**	**Primer Sequences** **Sense**	**Antisense**
FOXC2	5′-GAT CAC CTT GAA CGG CAT CT-3′	5′-ACC TTG ACG AAG CAC TCG TT-3′
CLIP4	5′-GCA TCA TGC CAG GAA ATT CT-3′	5′-TTT GTT GGA CCT GAG GAA CC-3′
PBRM1	5′-TGA TGG CCA ACA AGT ACC AA-3′	5′-AGA TCA AAG ACT CCG GCT CA-3′
SETD2	5′-TCA CAA GGC AGA CTC AGT GG -3′	5′-CTG CTG TCT TGG GCT TTT TC-3′
BAP1	5′-GCC TGA GGA GTC CAA GTC AG-3′	5′-CTG GAG GCT TCA CCA CTA GC-3′
KDM5C	5′-GTC ATT TGC AAC CCC TGA GT-3′	5′-AAT GGG ATG AGG GGT AAA GG-3′
AQP1	5′-CAA CTT CAG CAA CCA CTG GA-3′	5′-GTC GGC ATC CAG GTC ATA CT-3′
DDX11	5′-TCT CTT GGC TCC GTG ACT TT-3′	5′-TTT AGT CGG TCC ACC AGG TC-3′
BAIAP2L1	5′-GGC AGG AGA CCT GTG TTG AT-3′	5′-AGC CTG AGG AGT TCC AGA CA-3′
TMEM38B	5′-TCG GGA ATG AAG GAA GTG AC-3	5′-AGC CAT TCA TCA CCT TCT GG-3′
GAPDH	5′-CAG CCT CAA GAT CAT CAG CA-3′	5′-GGT GCT AAG CAG TTG GTG GT-3′

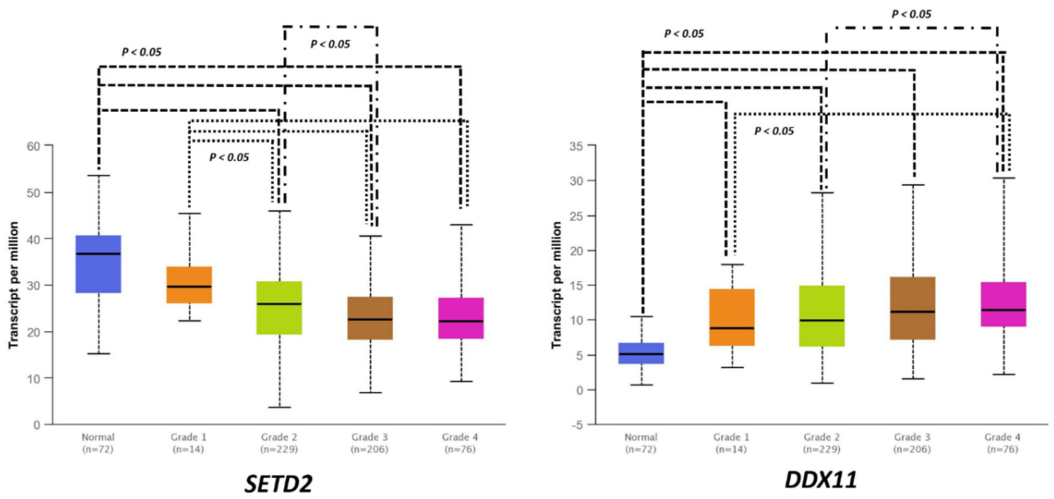

Figure S1. Validation of the expression of *SETD2* and *DDX11* in the TCGA database according to Fuhrman grades.

© 2020 by the authors. Licensee MDPI, Basel, Switzerland. This article is an open access article distributed under the terms and conditions of the Creative Commons Attribution (CC BY) license (http://creativecommons.org/licenses/by/4.0/).
